# *Vitis vinifera* L. Pruning Waste for Bud-Preparations as Source of Phenolic Compounds–Traditional and Innovative Extraction Techniques to Produce New Natural Products

**DOI:** 10.3390/plants10112233

**Published:** 2021-10-20

**Authors:** Dario Donno, Federica Turrini, Raffaella Boggia, Maddalena Guido, Giovanni Gamba, Maria Gabriella Mellano, Isidoro Riondato, Gabriele Loris Beccaro

**Affiliations:** 1 Department of Agriculture, Forestry and Food Science, University of Torino, Largo Braccini 2, 10095 Grugliasco, Italy; giovanni.gamba@unito.it (G.G.); gabriella.mellano@unito.it (M.G.M.); isidoro.riondato@unito.it (I.R.); gabriele.beccaro@unito.it (G.L.B.); 2 Department of Pharmacy, University of Genoa, Viale Cembrano 4, 16148 Genoa, Italy; turrini@difar.unige.it (F.T.); boggia@difar.unige.it (R.B.); 3 Azienda Agricola Geal Pharma, 10060 Bricherasio, Italy; info@gealpharma.it

**Keywords:** bud-derivatives, phenolics, phytochemical composition, spectroscopic analysis, chromatographic fingerprint, ultrasound extractions, antioxidant capacity

## Abstract

Herbal products are now considered among the most important sources of phenolic compounds: the FINNOVER project aimed at the creation and development of sustainable supply chains to extract and use natural biologically active agents. *Vitis vinifera* is one of the most utilised herbal products derived from buds and sprouts as polyphenolic food supplements for its homeostatic and astringent properties. This research was aimed to describe the antioxidant capacity and the phytochemical composition of *V. vinifera* herbal products by the application of spectroscopic and chromatographic fingerprints considering phenolics as potential markers to significantly differentiate traditional preparations (macerates) from innovative extracts obtained by an ultrasound extraction from *V. vinifera* buds. Two different commercial products were also considered. Flavonols were the most abundant class in ultrasound extracts (45%), while phenolic acids were the most important class in traditional macerates (49%) and commercial bud-preparations (about 50%). This study may support the potential use of *V. vinifera* bud-products (starting from pruning byproducts) as food supplements to integrate human diet with good amounts of phenolics. Finally, the use of different extraction methods on the same plant material could be an important development to produce innovative herbal products with a phytochemical composition similar to traditional preparations.

## 1. Introduction

Botanical products and food supplements derived from plants (e.g., herbal preparations) are available on the health-sector market and now widely appreciated by professionals and consumers. The interest in herbal supplements (containing botanicals) is greatly growing and the botanical market has increased in many countries, especially in Europe and North America. However, these products are still poorly studied, even if some in vitro/in vivo studies for veterinary and human use have been already reported [1,2,3]. Moreover, the high number of herbal products on the botanical market and an almost absence of specific legislation to control their quality and safety causes many issues on these products (e.g., adulteration, falsification, and fraud) [4].

Bud-preparations (or bud-derivatives) are a recent type of botanical preparation derived from plant material, which may be defined as homoeopathic medicines or food supplements based on national regulatory or commercial policies. Bud-derivatives are produced starting from the maceration of meristematic plant tissues (fresh material) in a mixed solution of solvents (ethanol, glycerol, and water) [5,6]. Herbal products derived from young sprouts and buds present many bioactive substances (defined as “botanicals”). These compounds with several health-promoting activities (e.g., alkaloids, amino acids, anthraquinones, coumarins, enzymes, glycosides, phenolic compounds, and terpenes) have been identified and characterised in several species [7]. Moreover, other health-promoting agents with positive effects in humans have been isolated in plant material and derived-preparations (e.g., micro- and macroelements, organic acids, nutritional substances, vitamins). Agronomic, environmental, genetic, and manufacturing traits may influence the concentration of these compounds [5]. Bud-preparations were studied in the FINNOVER project (innovative strategies for the development of cross border green supply chains), an Italy/France Interreg ALCOTRA cross-border project (period of activities: 2017–2020) aimed at the creation and development of sustainable supply chains to extract and use natural biologically active agents [8].

Even if the specific content of biologically active molecules in buds, in particular phenolics, was widely known since ancient times, bud-derivatives are still a “niche” product [9,10]. Polyphenolic compounds are mainly involved in plant development, and their production is influenced by biotic and abiotic stress condition [11]; vegetables, fruits, wine, coffee, and tea are the main sources for the human diet [12,13]. Polyphenols show a chemical structure based on aromatic rings with one or more hydroxyl groups that influence the anti-inflammatory and antioxidant properties of plant materials [14]. In the last few years, health-promoting benefits of phenolic supplementation have been reported in humans (e.g., against cardiovascular disease and ageing [15] and for the treatment of diabetes and obesity [16]). For this reason, the interest of the consumers is growing towards innovative plant foods and herbal supplements, which often show an even higher polyphenolic content [4]. One of the most utilised herbal products as polyphenolic food supplements for its health-promoting properties is *Vitis vinifera* bud-derivative [17,18]. It presents high contents of polyphenolic molecules and their amounts depend on the phenological stage [18]. Information on phenolics in berries, leaves, and seeds of the grapevine is widely available in the scientific literature [19,20], but little information is reported on the phenolic composition in buds [17,18]. Phenolic substances in *V. vinifera* may be divided into single phenolic molecules (e.g., hydroxycinnamic and hydroxybenzoic acids) and polyphenolic ones (e.g., flavonoids and stilbenes). In particular, flavonoids may include anthocyanins, catechins, and flavonols [18]. *V. vinifera* is reported in the scientific literature for its homeostatic and astringent properties in the treatment of several diseases (haemorrhoids, diarrhoea, bleeding, circulatory diseases, and varicose veins) [19,21].

The limited availability of young sprouts and buds in relation to the collecting time (between late winter and early spring) causes high costs to produce bud-preparations if compared to the other herbal products. Using the grapevine pruning wood could be an interesting way to turn a byproduct into an input for the bud-derivates production chain. The use of ecosustainable and innovative extraction techniques (e.g., ultrasounds) together with the traditional ones (e.g., cold maceration) may positively influence the herbal companies and the single producers with a high economic impact on the production yields [8]. Ultrasounds are now the most emerging green technologies [22] owing to the advantages shown in the different processing steps during the production of natural extracts (efficient and sustainable, relatively low-cost, green-effective) [23,24]. If the ultrasound-assisted extraction (UAE) is pulsed-mode utilised (PUAE), they are intermittently turned on and off during the extraction period to not degrade the thermolabile substances because a lower heat is produced if compared to a continuous-mode sonication [25].

This research was aimed to describe the phytochemical composition of *V. vinifera* herbal products considering phenolics as potential markers to significantly differentiate traditional preparations (macerates) from innovative extracts obtained by an ultrasound extraction from buds. Two different commercial products were also considered in comparison to the experimental preparations. Spectroscopic and chromatographic fingerprints were used to assess the quality and composition of these botanical products. Chromatographic profiles were obtained by HPLC methods to characterise the main polyphenols (e.g., flavonols, benzoic acids, catechins, cinnamic acids) as markers for their potential health-promoting activity. This study may support the potential use of *V. vinifera* bud-products as food supplements to integrate human diet with good amounts of phenolics resulting from their antioxidant and health-promoting properties. Finally, the use of different extraction methods (ultrasounds) on the same plant material could be an important development to produce innovative herbal products with a phytochemical composition similar to traditional preparations.

## 2. Results and Discussion

Phenolic profile (contents of benzoic acids, catechins, cinnamic acids, and flavonols) complemented by the measurement of the total polyphenolic content (TPC) and the antioxidant capacity (AOC) of the traditional macerates and ultrasound extracts, together to two commercial bud-preparations, of *V. vinifera* were defined by chemical analysis as already reported in similar studies [1,6,10,26].

### 2.1. Total Polyphenolic Content and Antioxidant Capacity

**Figure 1:**
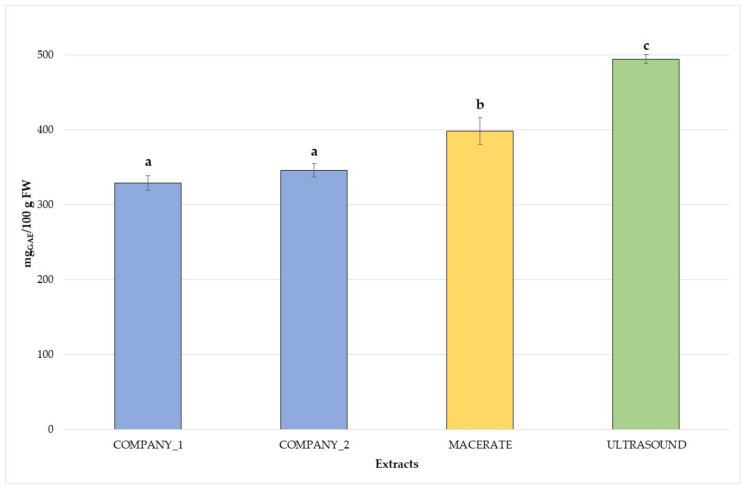
Total polyphenolic content of the analysed *V. vinifera* bud-extracts. Different letters for each extract highlight the significant differences at *p* < 0.05; blue—commercial bud-preparations, C1 and C2; yellow—traditional macerate, M; green—ultrasound extract, US.

**Figure 2:**
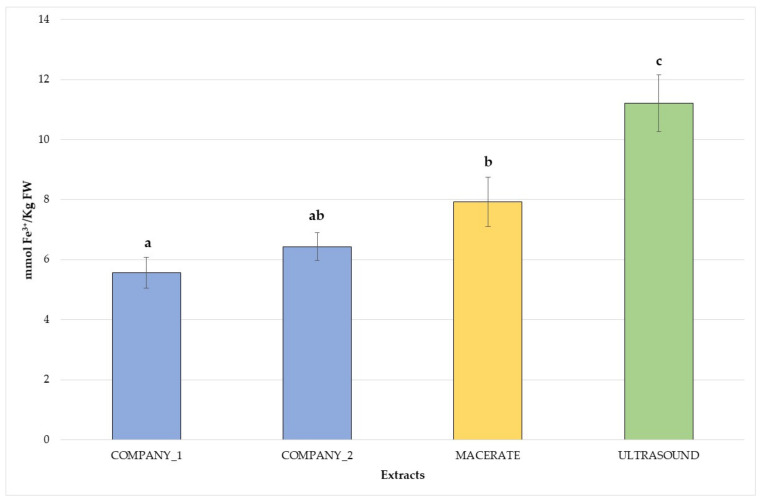
Antioxidant capacity of the analysed *V. vinifera* bud-extracts. Different letters for each extract highlight the significant differences at *p* < 0.05; blue—commercial bud-preparations, C1 and C2; yellow—traditional macerate, M; green—ultrasound extract, US.

Mean TPC and antioxidant capacity data are reported in Figure 1 and Figure 2. TPC values (Figure 1) ranged from 329.12 ± 9.35 mg_GAE_ 100 g^−1^ FW for commercial bud-preparations (C1) to 494.78 ± 5.93 mg_GAE_ 100 g^−1^ FW for the ultrasound extracts, in agreement with other studies on different species [4]. The highest polyphenolic content was observed for the ultrasound bud-extracts, but traditional bud-derivatives produced in this research also showed higher TPC values than commercial ones (more than 50–70 mg_GAE_ 100 g^−1^ FW). Significant statistical differences in antioxidant capacity (*p* < 0.05), expressed as FRAP assay, were observed among the bud-extracts with a trend very similar to the TPC levels. Antioxidant capacity varied from 5.56 ± 0.52 mmol Fe^+2^ kg^−1^ FW (commercial bud-preparation–C1) to 11.21 ± 0.94 mmol Fe^+2^ kg^−1^ FW (ultrasound extract–US), as shown in Figure 2, in agreement with previous studies on different species [4]. *V. vinifera* bud-derivatives resulted in potential sources of phenolics with good antioxidant capacity, as shown by similar studies on other species [10,27,28]. However, establishing the effect of every single phenolic compound to the antioxidant capacity may be very difficult due to the additive interaction and synergistic combination between the different substances and molecules (phytocomplex). Each antioxidant substance may improve the potential effectiveness of the other compounds, influencing the overall response [14]. This additive contribution may explain the statistical differences between the antioxidant capacity of the several analysed bud-extracts.

### 2.2. UV–Visible/Fluorescence Spectroscopy and HPLC Fingerprint

**Figure 3:**
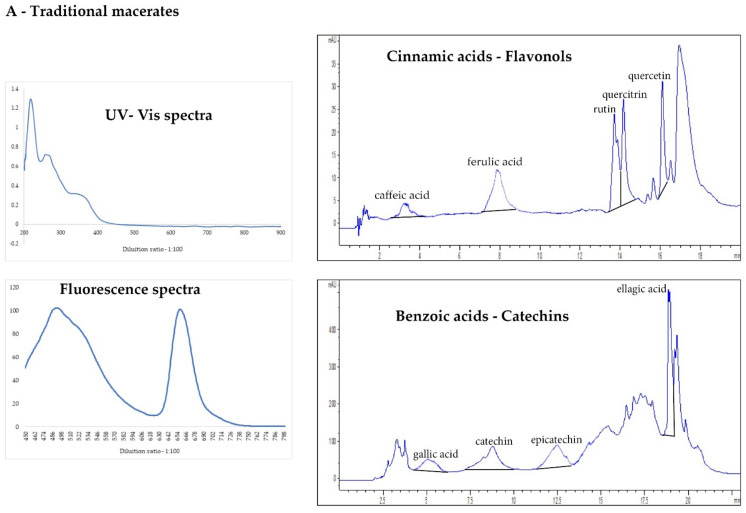

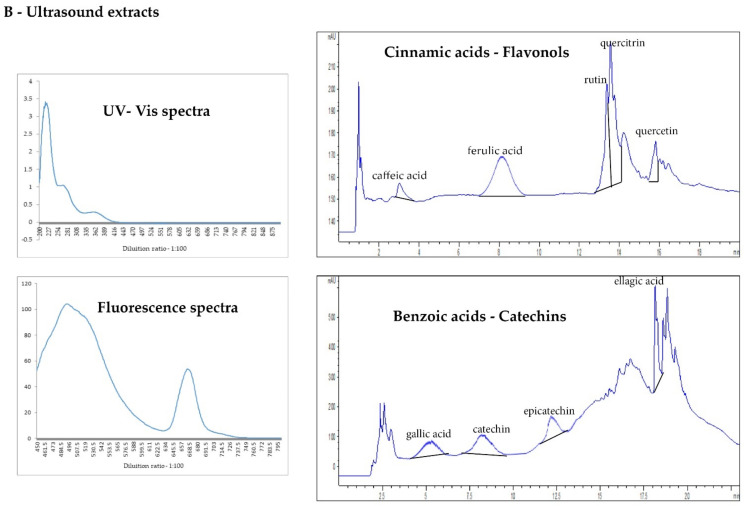
The chromatographic pattern and spectroscopic data of the analysed extracts: (**A**) traditional macerates and (**B**) ultrasound extracts.

The scientific literature on analytical strategies describes UV–Visible/Fluorescence spectroscopy and HPLC fingerprint as the most important characterization tools in natural product analysis [29,30,31]. This study showed a preliminary polyphenolic pattern of *V. vinifera* bud-macerates, commercial bud-preparations, and ultrasound extracts to compare bud-derivatives obtained with different extraction methods (maceration and ultrasounds). Figure 3 reports the phenolic chromatographic pattern and spectroscopic data of *V. vinifera* traditional and innovative bud-extracts.

As previously described in other studies [4], the pattern of absorbance in the UV–Visible region is strictly connected with the botanical origin of the plants. The intensity of the peaks is instead dependent on the total amount of polyphenolic compounds extracted [4]. As shown in Figure 3A,B, the spectral fingerprint of extracts obtained by the two different extraction methods (traditional and innovative) are similar, although at the same dilution the ultrasound extracts showed higher absorptions in the UV–Visible region, namely, a greater extraction of the phytocomplex compared to the corresponding traditional macerates.

In this research, the health-positive components were grouped into four phenolic classes to assess the contribution of all the classes to the polyphenolic phytocomplex composition of *V. vinifera* buds: cinnamic acids (as the sum of ferulic acid, coumaric acid, chlorogenic acid, and caffeic acid); flavonols (as the sum of rutin, quercitrin, quercetin, isoquercitrin, and hyperoside); benzoic acids (gallic and ellagic acids); and catechins ((-)epicatechin and (+)catechin). The identification and quantification of each phenolic substance are reported in Table 1.

**Figure 4:**
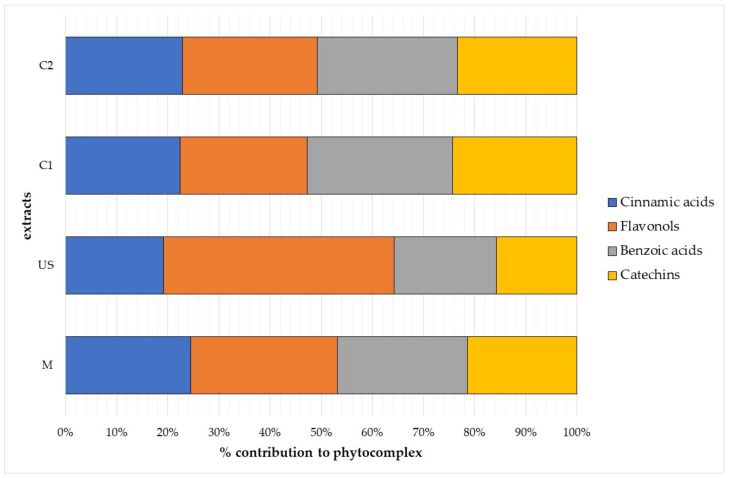
The phenolic composition of analysed *V. vinifera* bud-extracts. Mean values are shown (*n* = 3); commercial bud-preparations, C1 and C2; traditional macerate, M; ultrasound extract, US.

Data identified the analysed *V. vinifera* bud-extracts as an excellent source of polyphenols in relation to other similar preparations [2,4,8]. Contribution of each polyphenolic class to phenolic content is reported in Figure 4. Flavonols were the most abundant class in ultrasound extracts (45.1%), while phenolic acids (cinnamic plus benzoic acids) were the most important class in traditional macerates (49.8%) and commercial bud-preparations (50.9% for C1 and 50.3% for C2). Commercial bud-derivatives also showed a high content of catechins (about 24% for both products), but traditional macerates and ultrasound extracts presented a good amount as well (15–20% of the total phenolics).

Different markers for cinnamic acids were evaluated but not detected in all the extracts. Traditional macerates and ultrasound extracts presented similar amounts in cinnamic acids (242.30 ± 9.70 and 237.34 ± 6.08 mg/100 g_FW_, respectively), and ferulic acid was the most abundant (234.44 ± 9.51 mg/100 g_FW_ for traditional macerates and 227.43 ± 5.83 mg/100 g_FW_ for ultrasound extracts). In the last few years, several health-promoting functions and low toxicity of ferulic acid have been reported [32] for its cholesterol-lowering and free radical scavenging capacities, suggesting chemopreventive activity on cardiovascular diseases [33]. High contents of flavonols were also detected in *V. vinifera* bud-derivatives, in particular in ultrasound extracts (558.82 ± 22.68 mg/100 g_FW_), almost double the commercial extracts, confirming that these innovative preparations may be considered as a source of phenolics for dietary supplementation [4,10]. Flavonols are the main phenolics responsible for in vitro anticancer capacity (i.e., against lung, liver colon, and breast cancer) [34,35]. Commercial extracts, traditional macerates, and ultrasound extracts showed similar amounts in benzoic acids (about 250 mg/100 g_FW_); ellagic acid was the most abundant (222.35 ± 1.03 mg/100 g_FW_ for traditional macerates and 223.77 ± 1.35 mg/100 g_FW_ for ultrasound extracts) as already reported for other species in similar studies [4,8,10]. Benzoic acids, in particular ellagic acid, present many functional activities and biological capacity [36], including anti-inflammatory, antioxidative, antihepatotoxic, and anticancer properties [37,38]. All the *V. vinifera* bud-extracts analysed in this study resulted in an excellent source of catechins (about 200 mg/100 g_FW_), as shown in Table 1 (about 50 mg/100 g_FW_ for epicatechin and 150 mg/100 g_FW_ for catechin). Catechins show positive effects on humans owing to their antimicrobial, antidiabetic, anti-inflammatory, and antioxidant capacities [39]. The intake of food supplements rich in catechins may show a positive role in the prevention and treatment of several diseases (e.g., cardiovascular diseases), improvement of blood flow, elimination of several toxins, and inhibition of lipid peroxidation, cyclooxygenase enzymes, and human cancer cell line proliferation [40].

This study may support the potential use of *V. vinifera* bud-products as food supplements to integrate human diet with good amounts of phenolics. Indeed, all the analysed bud-extracts showed a superior content of polyphenolic compounds; in particular, innovative ultrasound bud-extracts presented a qualitative and quantitative phenolic composition similar to traditional bud-preparations (experimental bud-derivatives produced in the laboratory during the research and commercial products), even if ecosustainably and inexpensively produced in less time. For this reason, the use of different extraction methods (ultrasounds) on the same plant material may be an important development to produce innovative herbal products with a phytochemical composition similar to traditional preparations.

## 3. Materials and Methods

### 3.1. Plant Material

Buds were collected from *V. vinifera* (cv. Barbera), a wineyard in the Pellice Valley (Turin Province, Italy) during the spring (April–May 2019). The fresh buds were utilised by Geal Pharma (Bricherasio, Turin, Italy), an Italian commercial company, to formulate the relative bud-extracts in accordance with the procedure detailed in the French Pharmacopeia (Paris, France) [41]. The same plant material was utilised for the ultrasound extraction by PUAE. Two different Italian commercial products (reported as C1 and C2) were also considered in comparison to experimental preparations. The commercial products were supplied by a common pharmacy. They were among the bud-preparations most purchased by consumers according to the information provided by the pharmacist staff. No information on the cultivars used in their *V. vinifera* bud-extracts has been provided by the companies in the product labelling.

### 3.2. Preparation of Bud-Derivatives

#### 3.2.1. Traditional Maceration

The traditional maceration was carried out in accordance with the French Pharmacopeia (8th edition, 1965), in particular the detailed monograph “Homeopathic preparations” [41]. Raw material and a solution of glycerol and ethanol:water (95:5 *v/v*) were used to prepare the bud-extracts (ratio 1:20, dried weight). Biomolecules were cold macerated for 21 days and then filtered on filter papers (Whatman, Maidstone, UK, hardened ashless circles, 185 mm diameter), manually pressed, and filtered again after 2 days of decanting. Macerates (expressed as M) were dark stored at 95% relative humidity (R.H.), 4 °C, and normal atmosphere (N.A.) until analysis.

#### 3.2.2. Pulsed Ultrasound-Assisted Extraction 

Plant material was ground and then homogenized in a blender (Grindomix GM200, Retsch, Haan, Germany) at 5000 rpm (time: 20 s) and then sieved (150 μm mesh size). Moisture presented a value of about 84.50% performed by a MA Moisture Sartorius analyzer (Goettingen, Germany). All analyses were performed in triplicate and the results expressed as mean values (±standard deviations). PUAE was carried out directly by a sonicator (Hielscher UP200St, Teltow, Germany) composed by a titanium sonotrode (diameter: 7 mm) at a constant frequency of 26 kHz. The experimental conditions (amplitude: 30%; duty cycle: 70%; time of extraction: 20 min) were previously optimised on the same raw materials [4,10].

The extracts obtained (expressed as US) were filtered by Buchner (Whatman n. 1 paper), centrifuged (3000 rpm; 10 min), and dark stored at 4 °C until analysis.

### 3.3. Spectroscopic Analysis: Fluorescence and UV–Visible Fingerprint

#### 3.3.1. UV–Vis Spectroscopy

An Agilent Cary 100 spectrophotometer (Varian Co., Santa Clara, CO, USA) with 0.5 nm resolution was used to record UV–Visible absorption spectra (range: 200–900 nm); rectangular quartz cuvettes (1 cm path length) were utilised in the analysis at room temperature (25 ± 1 °C) [10]. Samples were diluted in the extraction solvent before the analysis to avoid signal saturation. Spectra were acquired in triplicate and then averaged. The extraction solvent was used as the blank standard solution.

#### 3.3.2. Fluorescence Spectroscopy

A PerkinElmer LS55B luminescence spectrometer (Waltham, MA, USA) was used to record the excitation-emission fluorescence spectra; a right-angle fluorescence spectroscopic technique was used to perform the analysis in triplicate at room temperature (25 ± 1 °C) [10]. A 10 mm SUPRASIL^®^ quartz cell (volume: 3.5 mL) by PerkinElmer (Waltham, MA, USA) and a standard cell holder were utilised. All the samples were excited at a fixed wavelength (430 nm) and the emission spectra recorded at 450–800 nm. The emission and the excitation monochromator slits were likewise set at 10 nm (speed: 600 nm/min). The 1:20 ratio with the solvent was used as the same dilution for each sample. For all the extracts, the emission spectra were averaged.

### 3.4. HPLC Sample Preparation and Chromatographic Analysis

All the samples were filtered with 0.45 μm pre-injection filters (polytetrafluoroethylene membrane, PTFE). Ultrasound extracts and macerates were analysed without dilution.

The analysis was carried out by using an Agilent 1200 HPLC-UV-Vis Diode Array Detector (Agilent Technologies, Santa Clara, CA, USA). The separation of phenolics was achieved on a Kinetex C18 column (4.6 × 150 mm, 5 μm, Phenomenex, Torrance, CA, USA). HPLC conditions are reported in Supplementary Materials (Table S1) following the previously validated methods, in accordance with Donno et al. [42].

In this research, four polyphenolic classes were evaluated and selected as markers for the evaluation of polyphenolic profile (phytocomplex): benzoic acids (gallic and ellagic acids), catechins ((−)epicatechin and (+)catechin), cinnamic acids (ferulic, coumaric, chlorogenic, and caffeic acids), and flavonols (rutin, quercitrin, quercetin, isoquercitrin, and hyperoside). Phytocomplex was defined as the sum of phenolics with antioxidant properties and health-promoting capacities; it was characterised by a comparison of retention times and spectroscopic data using standard molecules and the same experimental conditions. Compounds in the extracts were quantitatively determined by an external standard calibration method. The curves presented good linearity (peak area (y) vs. concentration (x) for a four-point plot). The phenolic profile was studied by the “multimarker approach” defined by Mok and Chau [43], intended as the extension of the “marker approach” (i.e., evaluating the amounts of one or very few markers or biologically active substances) because it utilised many specific molecules to study the phenolic profile of the preparation considered [44,45,46]. Each extract was analysed in triplicate and data were reported as mean value ± standard deviation to assess the repeatability of the methods.

### 3.5. Statistical Analysis

Analysis of variance (ANOVA) test for mean comparison with HSD Tukey multiple range test (*p* < 0.05) and Student’s t-test were carried out to highlight potential statistical differences in the phenolic composition between ultrasound extracts, experimental bud-derivatives, and commercial bud-preparations. Different letters highlighted statistically significant differences in the results with *p* < 0.05 (*n* = 3). SPSS 22.0 software was used for the statistical calculations.

## 4. Conclusions

In this research, an innovative approach to produce new *V. vinifera* herbal products was developed as an alternative to the common protocol (cold maceration for 21 days) to develop potential added-value preparations with an important impact for the vineyard growers and herbal/food supplement industries. The impact of the ultrasound extraction compared to the traditional extraction method was considered evaluating the total polyphenolic content, antioxidant capacity, and HPLC profile (13 markers with proved health-promoting properties for humans) of the most important phenolic compounds.

This research highlighted that the bud-derivatives produced by ultrasounds may be utilised as an excellent source of phenolics to be utilised as food supplements or other herbal products (e.g., cosmetics), representing an innovation in the sector of natural preparations. Results showed that *V. vinifera* ultrasound bud-extracts presented excellent amounts of health-promoting polyphenols, similar or even higher than commercial products, with a profile similar to the phytochemical pattern of the bud-derivatives obtained by a traditional cold maceration. Moreover, the ultrasonic extraction reduced the extraction times if compared to the traditional cold maceration (20 min vs. 21 days). The same approach, described for *V. vinifera* bud-extracts, may be also applied to other supply chains improving the total production systems.

Nevertheless, this is only preliminary research to provide alternative uses of *V. vinifera* pruning wood by using an ecosustainable time-saving extraction technology. Phytochemical studies by liquid chromatography coupled to mass spectrometry and biological/toxicological in vivo/in vitro tests are mandatory in the near future to confirm these preliminary results and the substantial equivalence of these innovative extracts in relation to conventional macerates.

## Figures and Tables

**Table 1:** HPLC fingerprint of the analysed *V. vinifera* bud-extracts.

		Caffeic Acid			Chlorogenic Acid			Coumaric Acid			Ferulic Acid					
Sample	ID	Mean Value	SD	Tukey Test	Mean Value	SD	Tukey Test	Mean Value	SD	Tukey Test	Mean Value	SD	Tukey Test			
COMPANY_1	C1	8.36	0.31	a	n.d.	/	/	n.d.	/	/	168.62	9.81	a			
COMPANY_2	C2	8.22	0.17	a	n.d.	/	/	n.d.	/	/	190.01	5.51	b			
MACERATE	M	7.86	0.19	a	n.d.	/	/	n.d.	/	/	234.44	9.51	c			
ULTRASOUND	US	9.91	0.25	b	n.d.	/	/	n.d.	/	/	227.43	5.83	c			
		**Hyperoside**			**Isoquercitrin**			**Quercetin**			**Quercitrin**			**Rutin**		
**Sample**	**ID**	**Mean Value**	**SD**	**Tukey Test**	**Mean Value**	**SD**	**Tukey Test**	**Mean Value**	**SD**	**Tukey Test**	**Mean Value**	**SD**	**Tukey Test**	**Mean Value**	**SD**	**Tukey Test**
COMPANY_1	C1	n.d.	/	/	n.d.	/	/	79.77	3.61	a	89.99	8.90	a	26.74	1.16	a
COMPANY_2	C2	n.d.	/	/	n.d.	/	/	91.23	3.74	a	105.95	5.86	ab	31.45	2.79	a
MACERATE	M	n.d.	/	/	n.d.	/	/	111.44	5.58	b	127.28	9.18	b	45.71	3.03	b
ULTRASOUND	US	n.d.	/	/	n.d.	/	/	106.86	8.71	b	356.10	9.85	c	95.86	4.12	c
		**Ellagic Acid**			**Gallic acid**			**Catechin**			**Epicatechin**					
**Sample**	**ID**	**Mean Value**	**SD**	**Tukey Test**	**Mean Value**	**SD**	**Tukey Test**	**Mean Value**	**SD**	**Tukey Test**	**Mean Value**	**SD**	**Tukey Test**			
COMPANY_1	C1	203.24	0.99	a	21.92	0.74	a	149.34	1.95	ab	42.37	2.59	a			
COMPANY_2	C2	208.09	3.59	a	29.16	11.30	a	157.25	2.02	b	44.99	1.38	ab			
MACERATE	M	222.35	1.03	b	29.42	0.69	a	145.13	5.15	a	67.67	2.85	c			
ULTRASOUND	US	223.77	1.35	b	23.59	0.53	a	145.37	3.90	a	50.11	1.81	b			

Different letters for each extract highlight the significant differences at *p* < 0.05. Results were reported as mg/100 g of fresh weight (FW). Commercial bud-preparations, C1 and C2; traditional macerate, M; ultrasound extract, US.

## Data Availability

Not applicable.

## Supplementary Materials

The following are available online at https://www.mdpi.com/article/10.3390/plants10112233/s1, Table S1: Chromatographic conditions of the used HPLC methods.
