# Supplementary material for: Vitis vinifera L. Pruning Waste for Bud-Preparations as Source of Phenolic Compounds–Traditional and Innovative Extraction Techniques to Produce New Natural Products
Source: Plants (Basel). 2021 Oct 20;10(11):2233. doi: 10.3390/plants10112233 (PMC8624332; doi:10.3390/plants10112233)
Supplement: Supplementary file 1 [file plants-10-02233-s001.zip › plants-1419917-supplementary.pdf]

plants

Supplementary Materials

Solvents, Chemicals, and Standards

Maceration and extraction solvents (ethanol and glycerol), analytical HPLC grade solvents (acetonitrile, methanol, and formic acid), reagents for HPLC buffer (potassium dihydrogen phosphate and phosphoric acid) were purchased from Fluka Biochemika (Buchs, Switzerland) and Sigma–Aldrich (St Louis, MO, USA). Cetyltrimethylammonium bromide (cetrimide) was purchased from Extrasynthése (Genay, France), while 1,2-phenylenediamine dihydrochloride (OPDA) was purchased from Sigma–Aldrich.

All polyphenolic standards (caffeic acid, chlorogenic acid, coumaric acid, ferulic acid, hyperoside, isoquercitrin, quercetin, quercitrin, rutin, ellagic acid, gallic acid, catechin, epicatechin, castalagin, vescalagin) were purchased from Sigma–Aldrich, while organic acids (citric acid, malic acid, oxalic acid, quinic acid, succinic acid, and tartaric acid) were purchased from Fluka Biochemika. Ascorbic acid and dehydroascorbic acid were purchased from Extrasynthése. Milli-Q ultrapure water was produced by Sartorius Stedim Biotech mod. Arium (Sartorius, Göettingen, Germany).

Stock solutions of cinnamic acids and flavonols with a concentration of 1.0 mg mL^{-1} were prepared in methanol. From these solutions, four calibration standards (1000 ppm, 50 ppm, 250 ppm, 125 ppm) were prepared by dilution with methanol; stock solutions of benzoic acids, tannins, and catechins with a concentration of 1.0 mg mL^{-1} were prepared in a solution of 95% methanol and 5% water. From these solutions, four calibration standards were prepared by dilution with 50% methanol-water. Stock solutions of organic acids with a concentration of 1.0 mg mL^{-1} were prepared in ultrapure water. From these solutions, four calibration standards were prepared by dilution with water. Finally, stock solutions of ascorbic and dehydroascorbic acids with a concentration of 1.0 mg mL^{-1} were prepared in methanol. From these solutions, four calibration standards were prepared by dilution with methanol.

Table S1. Chromatographic conditions of the used HPLC methods.

Method	Classes of Interest	Mobile Phase[1]	Wavelength (nm)
A	cinnamic acids, flavonols	A: 10 mM KH_2PO_4/H_3PO_4, pH = 2.8; B: CH_3CN	330
B	benzoic acids, catechins	A: $H_2O/CH_3OH/HCOOH$ (5:95:0.1 v/v/v), pH = 2.5; B: $CH_3OH/HCOOH$ (100:0.1 v/v)	280

[1]Elution conditions

A. gradient analysis: 5%B to 21%B in 17 min + 21%B in 3 min (2 min conditioning time); flow: 1.5 mL min^{-1}

B. gradient analysis: 3%B to 85%B in 22 min + 85%B in 1 min (2 min conditioning time); flow: 0.6 mL min^{-1}

C. gradient analysis: 5%B to 14%B in 10 min + 14%B in 3 min (2 min conditioning time); flow: 0.6 mL min^{-1}

D. isocratic analysis: a ratio of phase A and B: 95:5 in 10 min (5 min conditioning time); flow: 0.9 mL min^{-1}

Stationary phase: KINETEX – C18 column (4.6 × 150 mm, 5 µm)
